# An activator of phosphatidylinositol 3-kinase alpha restores cardioprotection from ischaemia/reperfusion injury in mice with coronary atherosclerosis or insulin resistance

**DOI:** 10.1093/cvr/cvaf111

**Published:** 2025-06-13

**Authors:** Pelin Golforoush, Elias Sulaiman, David He, Derek M Yellon, Sean M Davidson

**Affiliations:** The Hatter Cardiovascular Institute, University College London, 67 Chenies Mews, London WC1E 6HX, UK; The Hatter Cardiovascular Institute, University College London, 67 Chenies Mews, London WC1E 6HX, UK; The Hatter Cardiovascular Institute, University College London, 67 Chenies Mews, London WC1E 6HX, UK; The Hatter Cardiovascular Institute, University College London, 67 Chenies Mews, London WC1E 6HX, UK; The Hatter Cardiovascular Institute, University College London, 67 Chenies Mews, London WC1E 6HX, UK

**Keywords:** Ischaemia, Reperfusion, PI3K, Atherosclerosis, Mice

## Abstract

**Aims:**

In patients with coronary artery disease (CAD), morbidity and mortality from myocardial infarction remain high. Cardioprotective strategies such as remote ischaemic conditioning (RIC) are highly effective in animal models but have disappointed in large clinical trials. One explanation may be that ischaemia and reperfusion (I/R) experiments are typically conducted in mice that lack CAD. Unlike most mouse models, double-mutant SR-BI*ΔCT/ΔCT*;*Ldlr* KO mice do develop CAD when fed a high-fat diet (HFD). The aim of this study was therefore to use these mice to investigate cardioprotection in the setting of CAD. We hypothesized that RIC, which requires cell-surface receptor signalling, would be ineffective in these mice; but that UCL-TRO-1938, a phosphatidylinositol 3-kinase alpha (PI3Kα) activator that bypasses cell-surface receptors, would be cardioprotective.

**Methods and results:**

After 6-week HFD, double-mutant mice, but not wild-type (WT) or *Ldlr* KO mice, developed CAD as determined by histology. Anaesthetized mice were subject to 30-min coronary ischaemia and 2-h reperfusion. In line with our hypothesis, RIC reduced infarct size in WT and *Ldlr* KO mice, but did not reduce infarct size in double-mutant mice subject to I/R. We sought to understand the effects of CAD in double-mutant mouse hearts that might impair RIC, using RNA-sequencing (RNA-seq), immunostaining, and Western blot analysis. RNA-seq revealed significantly altered gene expression in double-mutant hearts compared with WT and *Ldlr* KO hearts, primarily in inflammatory pathways; in particular the interleukin-17 pathway. Coronary endothelial cells were activated, as shown by ICAM-1 expression in double-mutant but not *Ldlr* KO or WT. In contrast to RIC, treatment with UCL-TRO-1938 was cardioprotective in double-mutant mice.

**Conclusion:**

The double-mutant SR-BIΔCT/ΔCT;Ldlr KO mouse strain may be a more clinically translatable mouse model of I/R and cardioprotection. Furthermore, UCL-TRO-1938 is a promising cardioprotective drug as it remains effective in a mouse model with CAD.

## Graphical Abstract

**Graphical Abstract:**
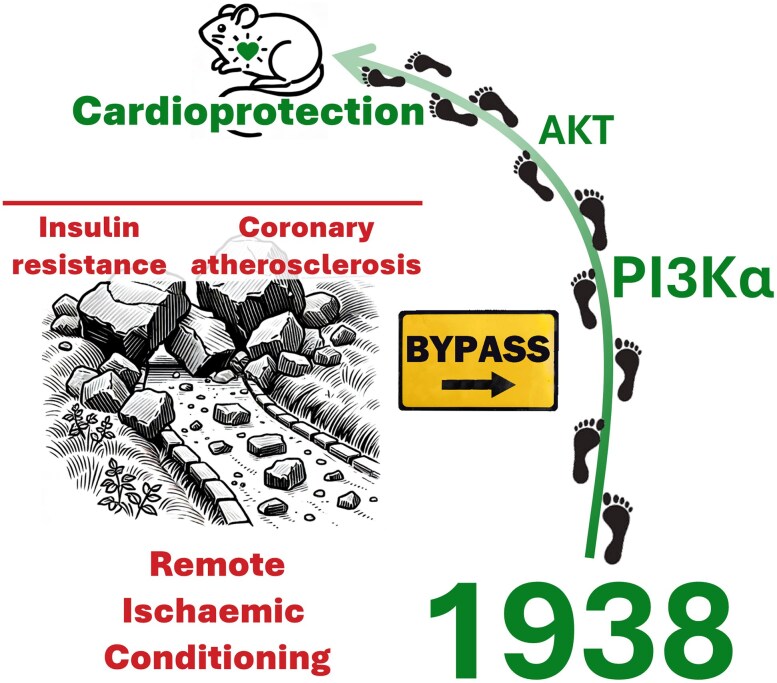

**Time of primary review: 42 days**

## 1. Introduction

Cardiovascular disease (CVD) is the leading global cause of death, with myocardial infarction (MI) and ischaemic stroke accounting for the majority of CVD-related deaths.^1^ ST-elevation MI (STEMI), often caused by plaque rupture and artery blockage, results in cardiomyocyte death due to ischaemia. Reperfusion is necessary to restore blood flow but causes additional irreversible cell death, called reperfusion injury.^2^ The final infarct size predicts long-term clinical outcome.^3^ Therefore, methods to minimize ischaemia and reperfusion (I/R) injury and reduce infarct size are vital to improve prognosis.^2,4,5^ Over the past 30 years, extensive research on reducing I/R injury has identified numerous promising approaches that effectively decrease infarct size in animal models. However, these strategies have yet to be successfully translated into patient care.^6^ One likely explanation for this is that animal experiments are typically conducted in young and healthy animals that lack the co-morbidities and co-medications commonly present in STEMI patients.^6–9^ This is particularly relevant given that diabetes mellitus is highly prevalent among STEMI patients, with nearly 30% of acute MI patients over the age of 55 having undiagnosed diabetes.^10^ Additionally, a study of apparently healthy individuals found that ∼20% were insulin resistant, a condition associated with early atherosclerosis.^11^ These findings underscore the importance of studying cardioprotection in diabetic and insulin-resistant animal models.

Notably, coronary atherosclerosis is present in nearly all MI patients^12^ and may impair key cardioprotective pathways, such as the reperfusion injury salvage kinase (RISK) pathway.^13,14^ The few studies of cardioprotection that have been conducted in atherosclerotic mice have returned inconsistent results.^8,13–17^ However, an important limitation of standard *ApoE* knockout (KO) and *Ldlr* KO atherosclerotic animal models is that, even when these mice are fed an atherogenic diet, plaques form predominantly in the aorta and great vessels and only rarely in coronary arteries.^18^ As such, these mice may not be optimal models for investigating the effect of coronary atherosclerosis on I/R injury. This leads us to suggest that the failure to translate interventions such as remote ischaemic conditioning (RIC) from animals, where it is highly effective, to patients, in whom large clinical trials have failed, may be partly attributed to the absence of coronary atherosclerosis in rodent models.^19–21^

In contrast to ApoE KO and Ldlr KO mice, which lack atherosclerosis in their coronary arteries, double-mutant SR-BI^ΔCT/ΔCT^;*Ldlr* KO mice (hereon called ‘DM mice’) containing a three amino-acid deletion at the C-terminus of SR-B1 and lacking *Ldlr* do develop atherosclerosis in their coronary arteries when fed an atherogenic diet.^22^ However, their response to I/R injury and cardioprotective strategies has not previously been investigated.

RIC is a simple intervention in cardiovascular research to protect against I/R injury.^23^ This approach involves subjecting a limb to short cycles of non-lethal ischaemia and subsequent reperfusion, which then protects the heart from subsequent I/R injury.^7,24^ The mechanisms underlying RIC are complex and involve pro-survival cellular signalling cascades that ultimately trigger the activation of endogenous protective mechanisms. The RISK pathway is defined as a group of conserved signal transduction pathways that are involved in RIC and induce cardioprotection when activated during early reperfusion.^25,26^ A major component of the RISK pathway is phosphatidylinositol 3-kinase (PI3K) leading activation of AKT and endothelial NOS (eNOS).^27^ Activation of this signalling cascade promotes cell survival by initiating a series of intracellular processes that prevent cell death.^27^ We and others have identified the PI3K/AKT pathway as being centrally involved in a range of approaches to protect the heart from I/R injury, and we further identified the alpha isoform of PI3K (PI3Kα) as centrally involved.^27–29^ Based on this concept, we recently developed a small molecule called UCL-TRO-1938 (hereon called ‘1938’) that selectively activates the alpha isoform of PI3K (PI3Kα) via a unique mechanism.^30^ Importantly, it bypasses cell surface receptors to enter cells and directly activate PI3Kα. We also showed that 1938 is cardioprotective in a commonly used model of myocardial I/R in wild-type (WT) mice.^30^ However, its efficacy in the setting of atherosclerosis has not yet been investigated. The aims of this study were therefore to investigate whether RIC and/or 1938 are capable of protecting mice with common co-morbidities from I/R injury. To this end, we used DM mice with coronary artery atherosclerosis, as well as mice that were insulin resistant. WT and *Ldlr* KO mice were used as controls.

## 2. Methods

### 2.1 Mice

A breeding pair of SR-BI*ΔCT/ΔCT*;*Ldlr* KO (Scarb1^tm1.1Okoch^ Ldlr^tm1Her^/J) mice was obtained from The Jackson Laboratory (Strain #:033709) and the colony maintained at UCL. SR-BI^ΔCT/ΔCT^ mice have a three amino-acid truncation at the C-terminus of SR-B1, which dramatically reduces hepatic levels of the receptor, while leaving fertility unaffected.^31^ For the insulin experiments, C57/Bl6 mice were purchased from Charles River. Animals were housed in individually ventilated cages, given food and water ad libitum, and exposed to a 12-h light–dark cycle. Animals received humane care in accordance with the UK Home Office Guide on the Operation of Animal (Scientific Procedures) Act 1986, project license PPL70/15358. All animal experiments were reviewed and approved by The University College London Animal Welfare and Ethical Review Board. Randomization was used, with the experimenter blinded to treatment, and the histological analysis of infarct size performed by the experimenter blinded to the treatment groups.

### 2.2 Diet

Mice were fed either normal chow or high-fat diet (HFD) chow containing 21% butterfat and 0.15% cholesterol (Envigo TD.88137) for 6 weeks (DM mice) and 9 weeks (C57/Bl6 mice) starting at 6 weeks of age. They were then culled, and hearts were collected for histology. For Western blot analyses, transcriptomic experiments, and infarct studies, DM mice were returned to normal chow for 1 week at the end of the 6-week HFD so that any change seen would be due to atherosclerosis rather than hyperlipidaemia *per se*.

### 2.3 *In vivo* I/R

Animals were anaesthetized with 100 mg/kg intraperitoneal sodium pentobarbital. The mice were intubated by tracheotomy and ventilated with room air using a small animal ventilator (MinVent, Type 845, Hugo Sachs Elektronik, Harvard Apparatus). The mice were then placed on a heating pad and the rectal temperature monitored and maintained at about 37°C. During the experiments, both the electrocardiogram (ECG) and heart rate were continuously recorded using PowerLab (ADInstruments). The chest was opened in the intercostal space between the third and fourth ribs to expose the heart, and a suture was placed around the left anterior descending (LAD) coronary artery followed by a snare to allow the occlusion and opening of the LAD. By tightening the suture snare to occlude the LAD coronary artery, the hearts were subjected to 30-min ischaemia, which was confirmed by both ST-segment elevation on the ECG and a change in heart colour. After 30 min, the snare was loosened, and the heart was allowed to reperfuse for the next 120 min. Mice were excluded if they did not survive the 120-min reperfusion part of the protocol. Mortality for *Figure 3B* was as follows: WT: 2 males and 1 female; LDLR KO: 3 males and 1 female; DM: no mortality and for *Figure 3D*, control: no mortality and 1938: 1 female.

### 2.4 Remote ischaemic preconditioning

RIC was performed on the anesthetized mice by placing a silicone cuff on the lower left limb and inflated to 200 mmHg to block the blood supply to the limb. After 5-min ischaemia, the cuff was deflated, and the blood supply was restored for 5 min. The cycle of I/R was repeated three times for each mouse just before the longer (30 min) ischaemic insult.

### 2.5 Drug administration

Insulin was administered to mice as a continuous infusion of glucose–insulin–potassium (GIK) solution, as described previously.^32^ The purpose of the glucose is to prevent the risk of hypoglycaemia due to insulin-stimulated glucose uptake, and the potassium reduces the risk of arrhythmias due to potential hypokalaemia.^32^ In vehicle-control mice, only glucose and potassium (GK) were infused. GK or GIK was administered via the jugular vein at 2.5 mL/kg/h (i.e.: 1 g/kg/h of glucose and 0.3 mmol/kg/h of KCl, with or without 0.3 units/kg/h of insulin) throughout the reperfusion period.

For infarct size studies in atherosclerotic mice, 1938 was dissolved in 100% DMSO, and 2 µL/g was slowly injected via the external jugular vein at a dose of 10 mg/kg, beginning 5 min before reperfusion. All mice in the infarction study received GK infusion (as described above) throughout reperfusion, to avoid potential hypoglycaemia or hypokalaemia that might result from the cellular uptake of glucose upon the activation of the PI3Kα pathway.

For Western blot analyses of hearts from DM mice, the mice were anaesthetized as above, and then 10 mg/kg 1938 or DMSO vehicle was administered as described. In all mice, GK was infused continuously (as described above) from the time of injection to termination. Fifteen minutes after drug administration, the hearts were collected, snap-frozen, and processed for the measurement of ICAM-1, PI3Kα, phospho-AKT, AKT, phospho-eNOS, eNOS, and β-actin by Western blot analysis.

### 2.6 Histology

For histological analysis of atherosclerotic plaques at the aortic root and coronary arteries, hearts were fixed in 4% paraformaldehyde overnight and then incubated successively in 10, 20, and 30% sucrose and then frozen in optimal cutting temperature compound (OCT); 10-µm sections were cut using a cryostat, taken at 100-µm intervals to visualize the coronary arteries. To visualize lipid content in the plaque, aortic root and whole heart sections (with coronary arteries present) were stained with Oil Red O and counterstained with haematoxylin. Images were taken using a microscope and camera, and atherosclerotic plaques in coronary arteries were quantified by manually counting Oil Red O-positive coronary arteries on three sections per heart. The average was reported for each mouse.

After 120 min of reperfusion, the chest was re-opened, and the heart was removed and canulated through the thoracic aorta with blood within the heart being washed out with saline. The LAD coronary artery was then re-occluded with the suture that had been left loosely in place following ischaemia, and the hearts were injected with 2% Evans blue to delineate the area at risk. These hearts were then frozen at −80°C for 10 min and subsequently cut into five to six slices of about 0.5-mm thickness. The heart slices were incubated in triphenyltetrazolium chloride (10 mg/mL) solution at 37°C, pH 7.4 for 15 min to delineate viable (stained red) from the necrotic tissue (white regions). Slices were then transferred to 10% formalin solution and fixed overnight. The heart slices, without right ventricular wall, were then scanned using a Cannon digital scanner. The total area of myocardium, the non-ischaemic area (stained with Evans blue), and the infarct area (the white area) of each slice were measured using ImageJ software. The area at risk was calculated by subtracting the non-ischaemic area (blue area) from the whole slice area and expressed as the percentage of the left ventricle. The infarct size was calculated as infarct area as a percentage of the area at risk.

### 2.7 Western blot analysis

Western blot analysis of tissue samples was performed as follows. At the end of the surgical procedure, the chest was opened, and the heart was removed and freeze-clamped in liquid nitrogen. Hearts were then homogenized in lysis buffer [100 mmol/L Tris-HCl, 300 mmol/L NaCl, 1% IGEPAL, pH 7.4 supplemented with protease inhibitors (cat 78438, Thermo Fisher Scientific) and phosphatase inhibitors (cat 78427, Thermo Fisher Scientific)] by disruption using a pestle and mortar and sonicated for 3 s on ice, five times. The supernatant was then collected, and after the addition of NuPAGE LDS sample buffer (4×) (Thermo Fisher Scientific), samples were boiled and stored at −80°C until SDS–PAGE was performed. Next, 20 µg of protein per well was loaded onto a 10% NuPAGE Bis-Tris gel (Invitrogen), resolved by SDS–PAGE, and transferred to PVDF membranes (Millipore) for Western blot analysis. Membranes were incubated with primary antibodies in 5% BSA/TBS–0.1% Tween-20 overnight at 4°C, washed three times for 10 min with TBS–0.1% Tween then incubated with appropriate fluorescent secondary antibodies in 5% BSA/TBS–0.1% Tween for 1 h, followed by washing three times for 10 min with TBS–0.1% Tween. Antibodies used are listed in *Table 1*. For AKT, antibodies were added simultaneously since they were from different species. For eNOS, the membrane was incubated first with anti-phospho-eNOS and imaged, and then the membrane was probed with anti-eNOS, for which the signal was much brighter. Fluorescence was visualized and quantified using an Odyssey Imaging System (Li-Cor Biosciences).

**Table 1:** Antibodies and dilutions used for Western blot analysis

Antibody	Dilution
Mouse anti-ICAM-1 (Thermo Fisher Scientific; MA5407)	1:250
Rabbit anti-phospho-AKT (S473) (Cell Signaling Technology; 9271)	1:1000
Mouse anti-(pan) AKT (Cell Signaling Technology; 2920)	1:1000
Rabbit anti-phospho-eNOS (Cell Signaling Technology; 9571)	1:1000
Rabbit anti-eNOS (Cell Signaling Technology; 9572)	1:1000
Rabbit anti-PI3K p110alpha (Cell Signaling Technology; 4249)	1:1000
Mouse anti-β-ACTIN (Santa Cruz; sc-47778)	1:2000
IRDye 680LT goat anti-mouse secondary antibody (Li-Cor Biosciences)	1:20 000
IRDye 800CW goat anti-rabbit secondary antibody (Li-Cor Biosciences)	1:15 000

### 2.8 Transcriptomics

Total RNA quality and quantity were assessed using an Agilent 4200 TapeStation with a Standard Total RNA assay. We processed 200 ng of total RNA per sample using the KAPA mRNA HyperPrep Kit (Roche KK8580) as per the manufacturer’s instructions. This process involved isolating mRNA with paramagnetic Oligo dT beads, fragmenting it via chemical hydrolysis, and synthesizing first-strand cDNA with Reverse Transcriptase and Actinomycin D. The second-strand cDNA was synthesized using dUTP instead of dTTP for labelling. Following A-tailing, the cDNA was ligated to xGen adaptors (IDT) and amplified using 12 PCR cycles with a high-fidelity polymerase. We verified high-yield, adaptor-dimer free libraries on the Agilent TapeStation 4200 using a High Sensitivity DNA 1000 assay. Sequencing was performed on an Illumina NextSeq 2000 at 800 pM, with a 56-bp single-read configuration plus 8-bp dual and unique molecular indices. Post-sequencing, data were processed using Illumina’s BCL Convert Software v3.75 to generate FASTQ files, which were then trimmed and aligned to the mouse UCSC mm10 genome using RNA-STAR v2.5.2b. Alignment quality was verified by Picard CollectRNASeqMetrics v2.8.1, ensuring minimal ribosomal RNA contamination. Reads were deduplicated with Je-suite v1.2.1, and transcript counts were obtained using FeatureCounts. Differential gene expression analysis was conducted using the BioConductor SARTools package with DESeq2. The RNA-seq data are available at Sequence Read Archive at NLM-NCBI under accession number: PRJNA1222332.

The gene count data were analysed in iDep 2.01 using Mouse genes GRCm39.^33^ Genes with minimum 0.5 CPM were kept for analysis. Counts were transformed for clustering and principal component analysis (PCA) using EdgeR: log2(CPM + c). First, the three genotypes were analysed (WT, *Lldr* KO, and DM). Significantly altered gene expression was identified using DESeq2 with FDR cut-off 0.05, minimum fold change 2, with filtering of lower counts. PCA indicated that DM samples clustered separately from WT and *Lldr* KO samples. Hierarchical clustering of the samples by Pearson distance using the top 2000 genes revealed that DM samples clustered separately from WT and *Lldr* KO samples (see Supplementary material online, *Figure S1*). Since there was no significant difference between WT and *Ldlr* KO (with the only differentially expressed gene being the X-linked Xist gene due to unbalanced sexes), in subsequent analyses, WT and *Lldr* KO groups were combined into a single group to increase the power in a two-group comparison with DM. Gene expression was re-analysed using DESeq2 as above, and these data were used for heatmap analysis, with DEGs sorted by fold change, with columns hierarchically clustered (*n* = 6 vs. 8). KEGG pathway analysis was performed using GSEA (pre-ranked fgsea), with significance cut-off FDR of 0.05.

### 2.9 Statistical analysis

Animals were randomly selected and researchers conducting data analyses were blinded to the genotype and to the diet. Sample size calculations for infarct size analysis were calculated using G*Power software^34^ using data from prior studies conducted by our team using the same methodology. Data analyses were conducted using ImageJ and GraphPad Prism v10. Results are presented as mean ± S.E.M. Results were compared using Student’s *t*-test for two groups or one-way analysis of variance (ANOVA) with Tukey post-test for three groups. RIC in mouse strains was compared using two-way ANOVA to examine main effects, with mouse genotype/diet and sham/RIC serving as independent variables. Upon detecting a significant overall effect, differences were assessed separately using Šídák post-hoc test. *P*-values < 0.05 were considered statistically significant.

## 3. Results

### 3.1 Atherosclerosis develops in the coronary arteries of double mutant mice

**Figure 1:**
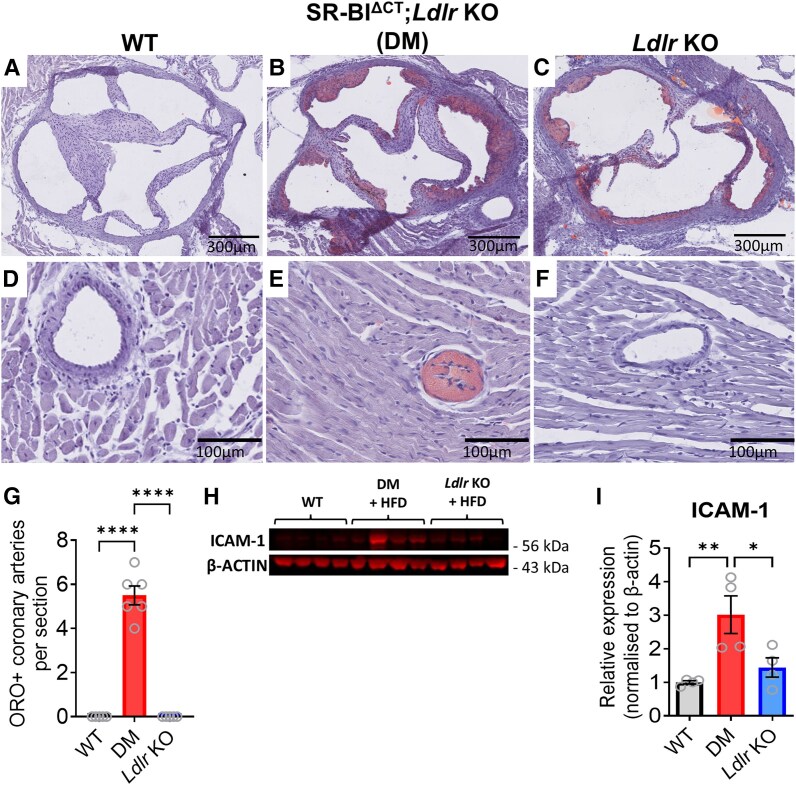
Characterization of hearts from WT, *Ldlr* KO and DM mice, after 6-week HFD. Oil-red O staining (ORO, red) with haematoxylin and eosin counterstain (purple) of heart cryosections showing aortic sinus (*A–C*) or coronary arteries and quantification (*D–G*). Western blot analysis of cardiac ICAM-1 protein levels in the mouse strains indicated (*H*), with quantification (*n* = 4) (*I*). Data are presented as mean ± SEM. *P < 0.05, **P < 0.01, and ****P < 0.0001 by one-way ANOVA and Tukey post-test.

A previous study by Fuller *et al*.^35^ established that SR-BI;*Ldlr* double-knockout mice fed a HFD for 12 weeks develop severe coronary atherosclerosis with some of the mice dying due to spontaneous MI. SR-BI^∆CT/∆CT^ mice have a milder phenotype than SR-BI KO.^31^ Furthermore, Shamsuzzaman *et al*. showed that SR-BI^∆CT/∆CT^/*Ldlr*-/- mice fed a Western diet for 26 weeks develop severe coronary atherosclerosis and exhibit high mortality rates, due to spontaneous plaque rupture with myocardial infarction and stroke.^22^ Since we were interested in developing a model of early atherosclerosis and wanted to avoid spontaneous infarcts from occurring and impacting the infarct study, we used SR-BI^∆CT/∆CT^/*Ldlr*-/- mice and fed them for a shorter period of 6-week HFD. We first verified that atherosclerotic plaques were detected in coronary arteries of DM mice after 6-week HFD, without evidence of spontaneous MI. After 6-week HFD, hearts of WT, DM and *Ldlr* KO mice were stained with Oil Red O. In WT mice fed the HFD, no plaques were observed in aortic sinuses or in the coronaries (*Figure 1A* and *D*). In *Ldlr* KO mice fed HFD, atherosclerotic plaques were observed exclusively in the aortic sinus but were absent from the coronary arteries, as expected (*Figure 1C* and *F*). In contrast, DM mice fed a HFD displayed plaques, not only in the aortic sinus (*Figure 1B*) but also in coronary arteries with ∼6 coronary arteries staining positive with Oil Red O per DM heart section (*Figure 1E* and *G*; Supplementary material online, *Figures S2A–D*).

Endothelial dysfunction is an early marker of atherosclerosis, with increased expression of adhesion molecules found on the surface of endothelial cells.^36,37^ The endothelium is also involved in certain cardioprotective manoeuvres.^38–40^ We therefore investigated ICAM-1 protein, a marker of endothelial dysfunction, and found it was increased in hearts from DM mice but not hearts from *Ldlr* KO mice, after 6-week HFD (*Figure 1H* and *I*).

### 3.2 Expression of RISK pathway components in double mutant mice

**Figure 2:**
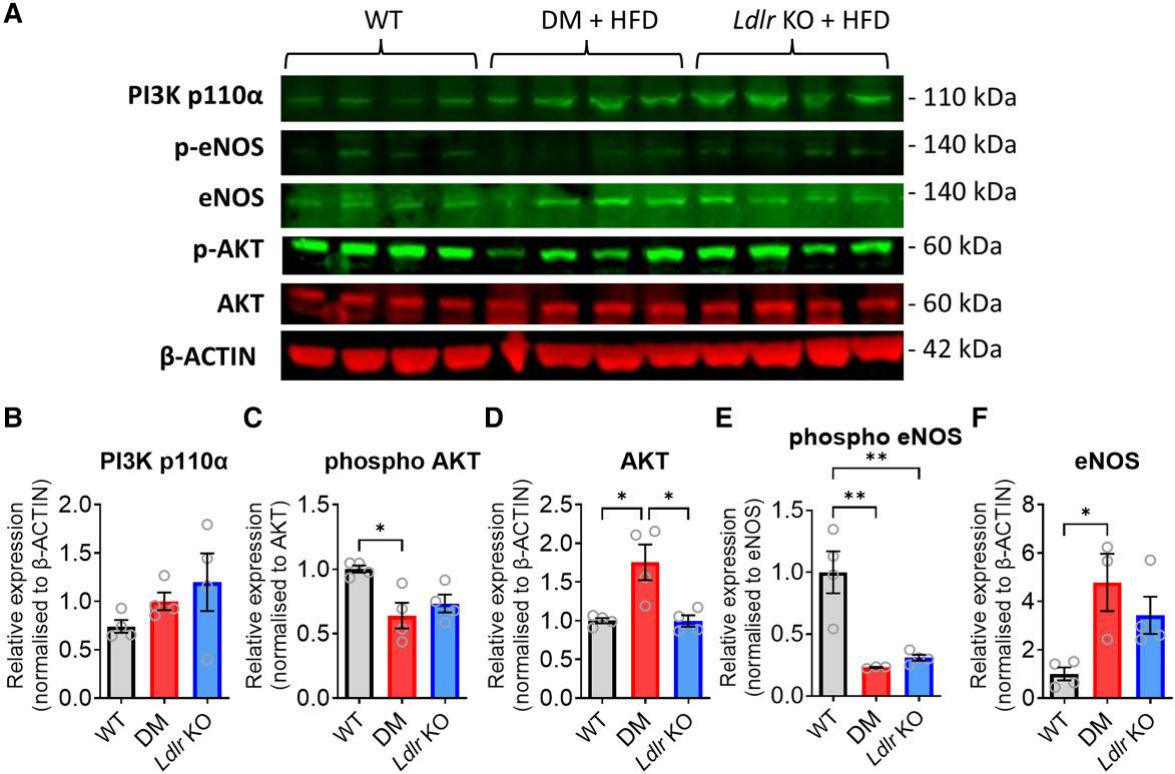
Western blot analysis (*A*) and quantification of myocardial p110α (*B*), phospho-AKT (*C*), total AKT (*D*), phospho-eNOS (*E*), and total eNOS (*F*) protein levels in the mice indicated (*n* = 4). Data presented as mean ± SEM. *P < 0.05 and **P < 0.01 by one-way ANOVA and Tukey post-test.

The PI3K/AKT pathway has been implicated in protection from numerous CVDs, including MI and heart failure,^41–43^ highlighting its significance in maintaining cardiomyocyte viability and cardiac homoeostasis. To assess whether this pathway may be a potential therapeutic target for cardioprotection in these mice, we investigated whether the major proteins in this pathway remained present in *Ldlr* KO and DM mice after feeding with HFD. Western blot analysis confirmed that p110α, the regulatory subunit of PI3K, which is also the target for 1938, was present in the hearts of all groups of mice (*Figure 2A* and *B*). Interestingly, there was significantly less phosphorylated AKT in hearts of DM mouse compared with those from WT mice (*Figure 2A* and *C*). Nevertheless, total levels of AKT were actually increased (*Figure 2A* and *D*). Similarly, eNOS, a downstream substrate of the PI3K/AKT and RISK pathway that is known to be involved in cardioprotection,^44^ was significantly decreased in DM mice in comparison with WT mice (*Figure 2A* and *E*), despite there being more total eNOS (*Figure 2A* and *F*). These data suggested that these central components of the RISK pathway are present and therefore remain a potential target for cardioprotection in DM mice. However, their basal activity (phosphorylation) is lower than in WT.

### 3.3 Remote ischaemic preconditioning is cardioprotective in WT and *Ldlr* KO but not in double mutant mice

**Figure 3:**
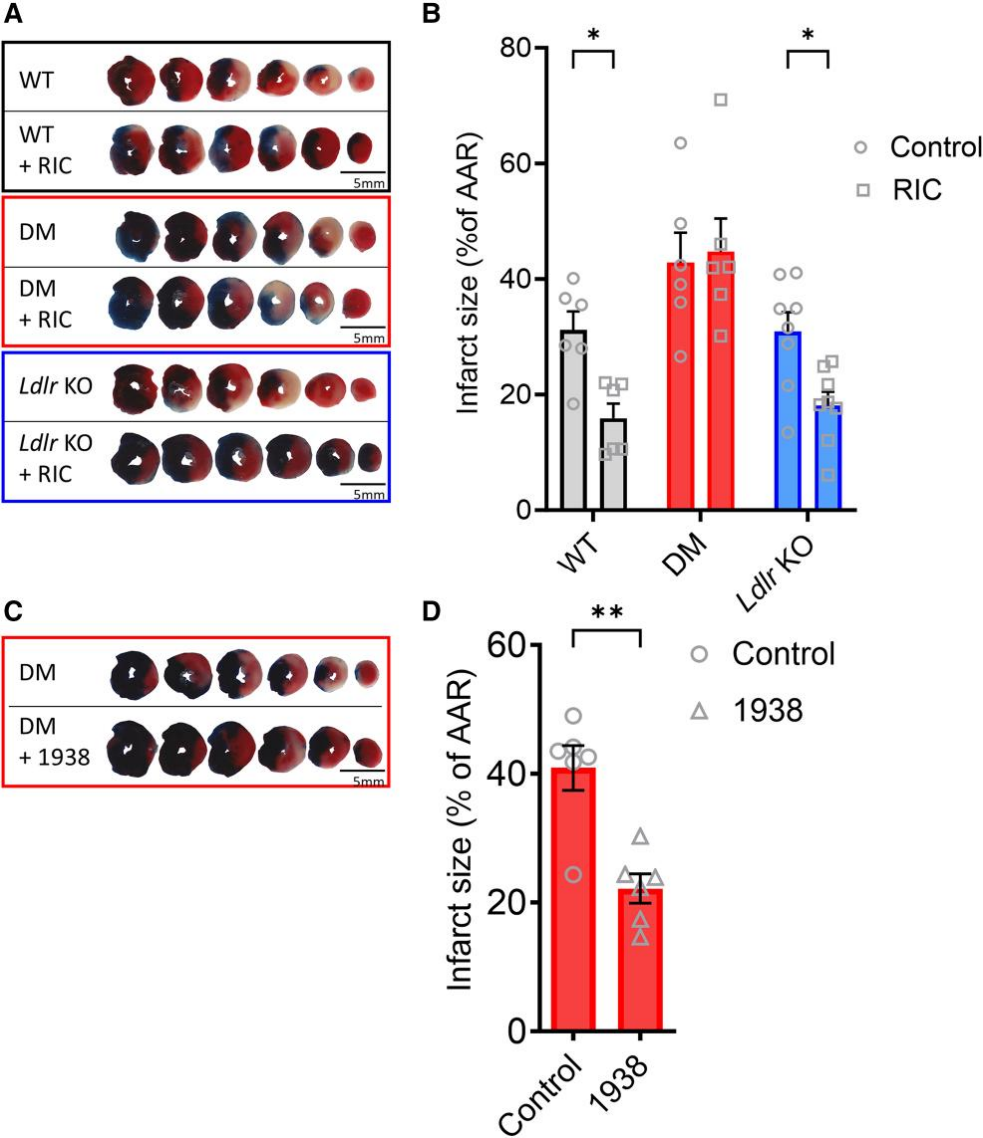
Remote ischaemic preconditioning (RIC □) is cardioprotective in WT and *Ldlr* KO but not in DM mice; 1938 (Δ symbol) is cardioprotective in DM mice. (*A–D*) Representative heart slices and quantification of infarct size relative to area at risk (AAR) in each treatment group (*n* = 6–8 per group). Data are presented as mean ± SEM. *P < 0.05 and **P < 0.01, by two-way ANOVA and Šidák post test (*B*) or Student’s *t*-test (*D*).

RIC has emerged as a promising strategy for protecting the heart against I/R injury by activating endogenous protective pathways. We subjected WT, *Ldlr* KO, and DM mice to I/R, with or without preceding RIC applied to the hind limb. The size of the ischaemic area at risk was similar in all groups (see Supplementary material online, *Figure S3A*). As expected, RIC was cardioprotective in the HFD-fed WT mice, markedly reducing myocardial infarct size following I/R (*Figure 3A* and *B*). RIC was also effective in HFD-fed *Ldlr* KO mice, limiting infarct size following I/R (*Figure 3A* and *B*). In contrast, RIC was ineffective in HFD-fed DM mice (*Figure 3A* and *B*), suggesting an impairment in the cardioprotective mechanisms elicited by RIC in the context of coronary atherosclerosis.

Importantly, in control experiments, RIC remained effective in DM mice on normal chow that were subject to I/R (see Supplementary material online, *Figure S4A–C*). Therefore, the lack of cardioprotection seen in HFD-fed DM mice is not due to the genetic mutations *per se*, but is due to the coronary atherosclerosis that develops in these mice when fed HFD.

### 3.4 1938 is cardioprotective in double mutant mice

We have previously shown that 1938 administered at reperfusion is cardioprotective in WT mice.^30^ We therefore investigated whether it would be cardioprotective in DM mice subjected to I/R. In DM mice, 1938 significantly reduced myocardial infarct size compared with vehicle, as evidenced by histological analysis (*Figure 3C* and *D*). The AAR was similar in both groups (see Supplementary material online, *Figure S3B*). Thus, 1938 is cardioprotective in the presence of coronary atherosclerosis as found in DM mice.

**Figure 4:**
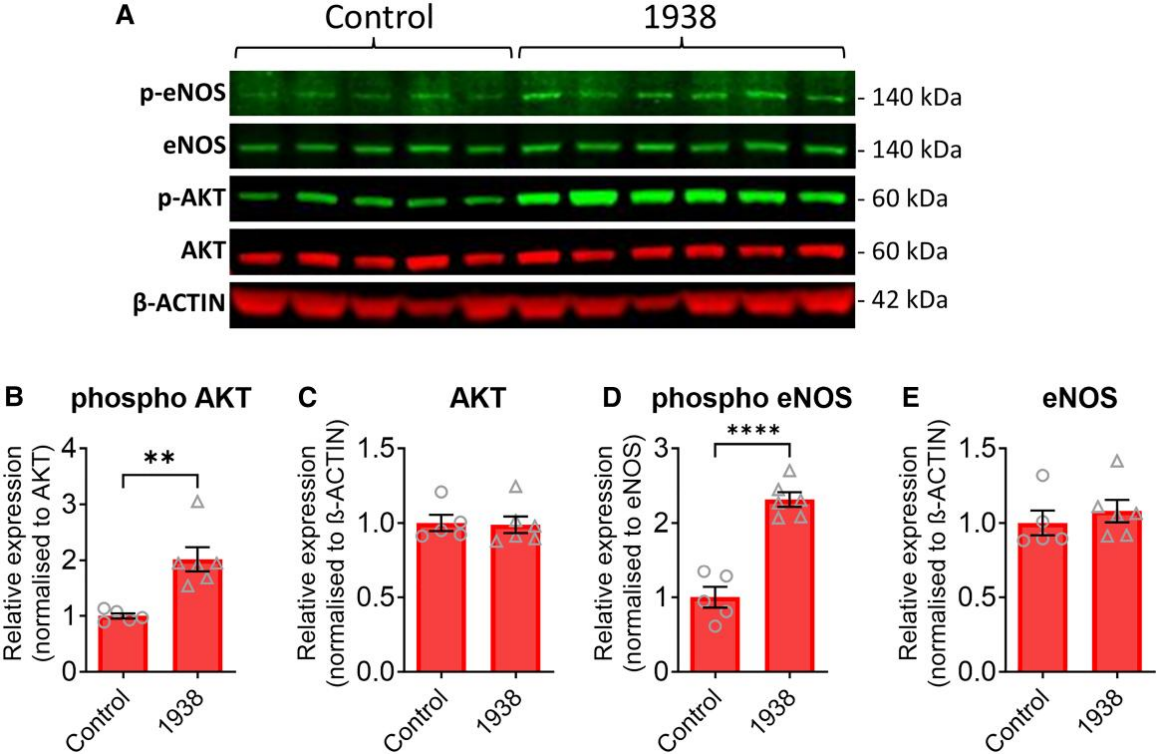
PI3Kα activator 1938 (Δ symbol) induces phosphorylation of AKT and eNOS in hearts of DM mice. Western blot analysis (*A*) and quantification of cardiac phospho-AKT (*B*), total AKT (*C*), phospho-eNOS (*D*), and total eNOS (*E*) protein levels in DM mice (after 6-week HFD and 1-week normal chow) administered vehicle or 1938 for 15 min. Data are depicted in the graph as a fold change (*n* = 5–6). Data are presented as mean ± SEM. **P < 0.01 and ****P < 0.0001 vs. untreated DM mice by Student’s *t*-test.

Mechanistically, 1938 mitigates myocardial injury by activating the PI3Kα/AKT pathway implicated in cardioprotection.^27,30^ We confirmed that 1938 treatment increased the phosphorylation of both AKT (*Figure 4A* and *B*) and eNOS (*Figure 4A* and *D*) in DM mice. The total levels of these proteins were unchanged (*Figures 4A*, *C*, and *E*).

### 3.5 Insulin-mediated cardioprotection is defective in insulin-resistant mice, but 1938 is effective

**Figure 5:**
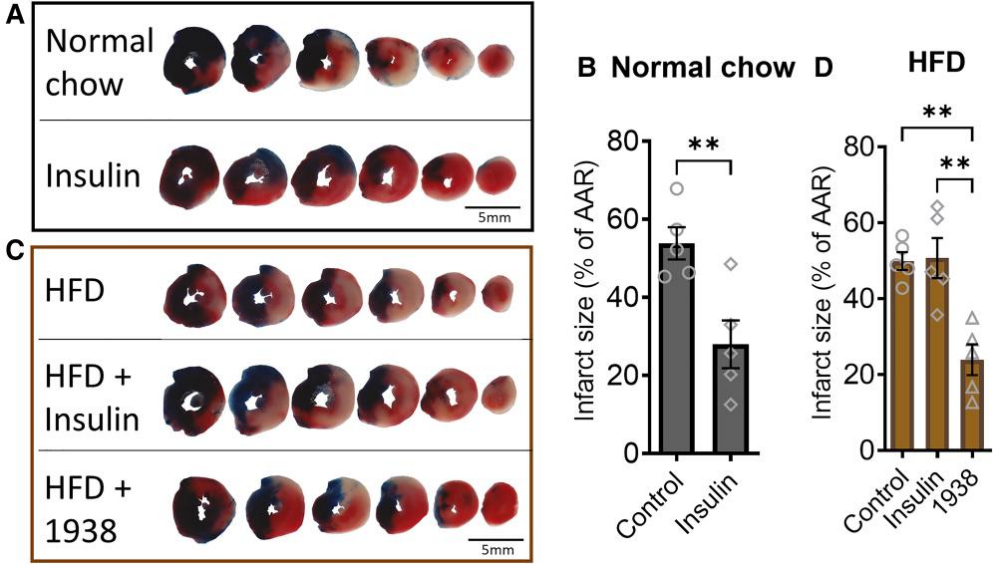
Insulin (◊) is cardioprotective in mice fed normal chow but not in insulin-resistant (HFD-fed) mice; 1938 (Δ) is cardioprotective in insulin-resistant mice. (*A–D*) Representative heart slices and quantification of infarct size relative to area at risk (AAR) in each treatment group (*n* = 5 per group). Data are presented as mean ± SEM. **P < 0.01, by Student’s *t*-test (*B*) or one-way ANOVA and Tukey post-test (*C*).

Since 1938 bypasses cell-surface receptors to activate the RISK pathway by directly binding PI3Kα, we hypothesized that, in contrast to insulin, 1938 would remain cardioprotective in the setting of insulin resistance. We first verified that insulin (administered in the form GIK) was cardioprotective in mice on normal chow subject to IR (*Figure 5A* and *B*), and furthermore, that it was no longer cardioprotective in insulin-resistant mice (*Figure 5C* and *D*). Importantly, however, we demonstrated that 1938 was effective in reducing infarct size in the insulin-resistant mice (*Figure 5C* and *D*). The size of the ischaemic area at risk was similar in all groups (see Supplementary material online, *Figure S3C* and *D*).

### 3.6 mRNA expression is altered in double mutant hearts

**Figure 6:**
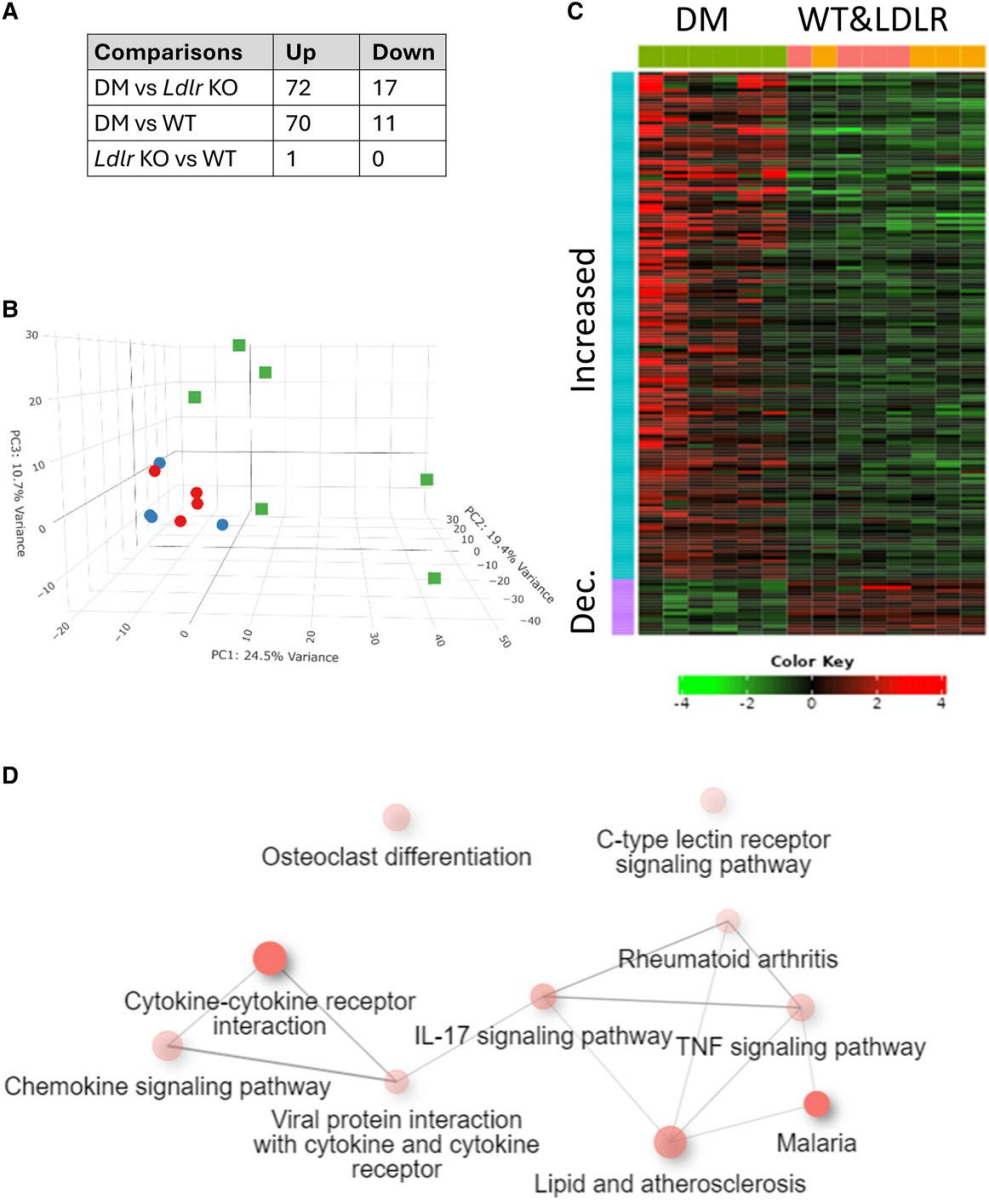
RNA-seq analysis of hearts from mice following HFD. (*A*) The number of genes with increased or decreased expression between different groups. WT and *Ldlr* KO are the same apart from sex-specific *Xist* gene. (*B*) PCA of gene expression in WT (blue circles), *Ldlr* KO (red circles), and DM hearts (green squares). (*C*) Heat map of genes with significantly increased (red) or decreased (green) expression levels in DM hearts in a two-group comparison with WT and *Ldlr* KO mice, sorted by fold change, with columns hierarchically clustered (*n* = 6 vs. 8). (*D*) Significantly altered KEGG pathways in DM hearts in comparison with WT and *Ldlr* KO hearts.

Since RIC was effective in WT and *Ldlr* KO, but not in DM mice, we investigated whether the presence of coronary atherosclerosis in the DM hearts might have altered gene expression in pathways that affect the induction of cardioprotection. By comparing gene expression profiles between WT and DM mice hearts, we identified specific genes and pathways that were altered in DM mice, indicative of underlying molecular changes associated with coronary atherosclerosis. We observed a significant up-regulation of 70 genes and down-regulation of 11 genes in DM mice compared with WT mice (*Figure 6A*; Supplementary material online, *Table S1*). By PCA, WT and *Ldlr* KO groups clustered together, separately from the DM heart (*Figure 6B*). Since no differentially expressed genes were identified between WT and *Ldlr* KO heart (except for *Xist* due to an imbalance in number of males/females), these two groups were combined and re-compared with DM in order to increase the statistical power. This reanalysis identified 154 genes with increased expression and 17 with decreased expression in DM hearts (*Figure 6C*). Following gene ontology analysis, pathways affected in DM mice were found to be enriched in processes related to chemokine, interleukin (IL)-17, and tumour necrosis factor (TNF) signalling pathways (*Figure 6D*; Supplementary material online, *Table S2*).

The expression level of certain ncRNAs was significantly altered in DM hearts, in comparison with WT and *Ldlr* KO hearts (see Supplementary material online, *Table S1*). In particular, expression of the vasculoprotective CARMN lncRNA^45^ was decreased by ∼58% (*P*_adj_ = 1 × 10^−7^). Loss of the Charme lncRNA causes myogenic defects and heart remodeling,^46^ and this was found to be ∼54% lower (*P*_adj_ = 0.01) in DM mice. Lastly, the MeXis lncRNA, which is involved in macrophage cholesterol efflux and atherogenesis,^47^ was increased by ∼3-fold (*P*_adj_ = 8 × 10^−6^).

## 4. Discussion

One of the main conclusions from our study is that the presence of coronary atherosclerosis in DM mice impairs cardioprotection by RIC. This observation may partly explain the disconnect between animal studies in which RIC is usually effective,^19^ and large clinical trials of RIC in STEMI patients, which are mostly neutral.^48,49^ Of note, nearly all (i.e. ∼95%) patients who experience acute MI have coronary atherosclerosis.^12,50,51^ We propose that the ineffectiveness of RIC may be a consequence of either the presence of *coronary* endothelial dysfunction (which was confirmed by ICAM-1 expression), altered cardiac gene expression, or other cardiac consequences of atherosclerosis (such as inflammation), which were present in the DM mice but absent from the *Ldlr* KO mice. A corollary of the above conclusion is that standard atherosclerotic mouse models such as *Ldlr* KO mice might not be optimal for studying cardioprotective strategies against I/R injury.

In contrast to RIC, 1938 was cardioprotective in DM mice, to a similar extent as we previously found in WT mice.^30^ We interpret this result as indicating that compounds such as 1938 that are able to bypass cell-surface receptors and/or dysfunctional endothelium, and directly activate PI3Kα in the myocardium, might be more effective in the clinical scenario. Although often overlooked in I/R studies, coronary atherosclerosis is a critically important factor, since it may impair signalling via the RISK pathway.^13,14^ Similarly, in the setting of insulin resistance, insulin receptor substrate 1 is down-regulated, impairing downstream signalling.^52^ As has been previously noted, this oversight may explain the failure to translate interventions such as RIC from animals, where it is highly effective, to patients.^8,21^ Endothelial activation and dysfunction occur at an early stage in the development of atherosclerosis.^53^ Furthermore, subclinical atherosclerosis is associated with early insulin resistance, even in normoglycaemic low-risk individuals.^54^

Previous studies have investigated the efficacy of cardioprotective interventions in mice with atherosclerosis, using standard genetic models in which the plaques are located primarily in the aorta and not in the heart (reviewed in Golforoush *et al*.^8^). For example, RIC and anaesthetic-induced preconditioning were found to be effective in *Ldlr* KO mice that had been fed a HFD.^55^ However, atherosclerosis was not assessed in this study and is usually absent from the coronary arteries of *Ldlr* KO mice. Shamsuzzaman *et al*.^22^ recently reported that mice with a C-terminal mutation in *Srbi* and also lacking *Ldlr* develop coronary atherosclerosis when fed a Western diet for 26 weeks. Interestingly, the level of myeloperoxidase was increased, and treatment with an inhibitor of myeloperoxidase *throughout* the entire 26 weeks reduced the incidence of spontaneous MI and stroke in these mice.^22^ However, the authors did not investigate whether a treatment applied at reperfusion could reduce infarct size. In our study, we saw no change in myeloperoxidase gene expression in DM hearts.

Some previous studies have examined the effect of hypercholesterolaemia on I/R in rabbits, which develop atherosclerosis in response to HFD. However, in this model, the data on the effectiveness of cardioprotective strategies such as RIC are mixed.^13,14^ In most HFD experiments, animals are fed HFD continuously and are not returned to normal chow before experiments—they are therefore still hyperlipidaemic, which, in itself, it known to affect cardiac vulnerability to I/R injury.^56^ Of note, although hyperlipidaemia is clearly a risk factor for atherosclerosis, atherosclerosis can occur in ∼40% of individuals with normal lipid levels.^57^ Therefore, in order to examine the effect of atherosclerosis, we returned mice to normal chow after HFD for a defined recovery period prior to I/R. Importantly, the impairment in RIC was not due to HFD feeding protocol itself, because RIC remained effective in a standard model of aortic atherosclerosis in *Ldlr* KO mice fed HFD. Furthermore, the ineffectiveness of RIC was not due to the absence of the *Ldlr* gene or the mutation of *Scarb1* gene encoding SR-BI but was only apparent when DM mice were fed the HFD protocol, inducing coronary atherosclerosis.

RIC is believed to induce cardioprotection via both a humoral arm in which a blood-borne factors/signals from the limb to the heart and a neural arm via vagal signalling to the spleen.^58^ It is conceivable that coronary atherosclerosis impairs either or both of these mechanisms. The identity of the blood-borne factor/s is not known, although various peptides and factors have been proposed.^38,59,60^ A common aspect of many of these factors is that they activate pro-survival signalling pathways (e.g. the so-called ‘RISK’ pathway or ‘SAFE’ pathway) via cell-surface receptors. Thus, RIC signalling may potentially be disrupted in the setting of atherosclerosis as a consequence of endothelial inflammation and dysfunction. Without knowing the precise mechanism of RIC, it is difficult to establish the exact reason that RIC fails in the DM mice.

Interestingly, endothelial dysfunction is a common underlying factor for many of the risk factors associated with CVD, including diabetes, smoking, age, and diet. We have previously shown that diabetes and age impair cardioprotection in both *in vivo* animal models^61^ and *ex vivo* studies with human cardiac muscle.^62^ This suggests that endothelium dysfunction may be a common factor underlying the impaired cardioprotection seen in the presence of co-morbidities, although further studies are required to confirm this.

There is a close reciprocal relationship between endothelial dysfunction and insulin resistance.^63^ As noted in the introduction, insulin resistance is widely prevalent among STEMI patients, affecting both those with diagnosed and undiagnosed diabetes. Furthermore, both AMI and surgery itself can induce transitory insulin resistance, driven primarily by the surgical-stress response, which triggers hormonal, inflammatory, and autonomic changes that interfere with normal insulin signaling.^64^ In addition, Type 2 diabetes and insulin resistance increase the risk of STEMI, are prevalent in STEMI patients, and worsen outcomes.^65^ This may explain why the results of most clinical trials of insulin in cardioprotection have been equivocal.^32,66^ Indeed, we observed that, while insulin reduced infarct size in WT mice subject to I/R, insulin-resistant mice were not protected. Importantly, 1938 was able to bypass this signalling defect and protect the hearts of insulin-resistant mice.

We observed altered levels of cardioprotective kinases in experimental mice at baseline (i.e. prior to I/R). The levels of total AKT and eNOS were significantly increased only in DM mice, while both phospho-AKT and phospho-eNOS were decreased in *Ldlr* KO and DM mice. A possible explanation for lower baseline AKT phosphorylation in the DM mice is insulin resistance, which may have been induced by the HFD feeding. For instance, cardiac lipid deposition inhibits insulin signalling by increasing of IRS phosphorylation.^67,68^

There were significant changes in gene expression in the hearts of DM mice. Gene ontology revealed that the majority of these involved pathways related to inflammation, including TNFα and IL-17 signalling. The role of IL-17 in atherosclerosis is complex and context dependent, with the potential to both promote atherosclerosis by increasing inflammation and improve atherosclerosis by promoting tissue repair roles.^69^ Interestingly, Il-17 has been shown to contribute to I/R injury by regulating cardiomyocyte apoptosis and neutrophil infiltration.^70^ We speculate that 1938 may protect against IL-17-mediated cardiotoxicity, but this will require experimental investigation. Increased pathways involved in lipids and atherosclerosis likely relate to the formation of foam cells (e.g. genes *Olr1*, *Abca1*, and *Abcg1*). Increased chemokine signalling, including *Ccl2(Mcp1)*, *Ccl7(Mcp3)*, *Ccl12(Mcp5)*, and *Ccr3*, is likely involved in the recruitment of monocytes and macrophages. Increases in immune cell signalling proteins (e.g. *vav1*, *Hck*, *Ncf4*, *Ptpn6*, *Rac2*, and *Trem2*) are likely related to neutrophil activation. Increases in acute phase response (AP1 transcription factors and *Egr*) reflect inflammation, cell proliferation, and the response to vascular injury. Overall, the enrichment in pathways associated with inflammatory processes in DM mice suggests a heightened pro-inflammatory milieu in DM mice. While there is some evidence in the literature suggesting that RIC can attenuate inflammation, further studies are required to understand the impact that a pro-inflammatory environment might have on RIC and cardioprotection.^71^

A limitation of this study is that both male and female mice were combined for the analyses. Future studies will be conducted to determine whether there are sex-specific differences. It is possible that differences in cardiac gene expression would be detected if the number of replicates in RNA-seq experiments was increased further. Furthermore, since RNA-seq was conducted on bulk tissue samples, cell-type-specific information is not available. Future studies employing single-cell RNA-seq will be necessary to dissect the specific contributions of individual cell types to the mechanisms identified here.

Finally, it is worth noting that other reasons are likely to have contributed to the lack of success with RIC in large clinical outcome clinical studies in STEMI, as has been widely discussed. These include the relatively low event rates (reducing statistical power), the high prevalence of other co-morbidities that may impair cardioprotective strategies, and the presence of ‘background’ therapy in STEMI patients, which may impart some degree of protection.^5,6,9^

In conclusion, the presence of coronary atherosclerosis and insulin resistance impedes cardioprotection by RIC. However, activation of the RISK pathway by 1938 remains cardioprotective in this setting. We propose that mice with coronary atherosclerosis may be a more suitable experimental model that better reflects the STEMI patient population.^8^

**Translational perspective**

Remote ischaemic conditioning (RIC) reduces ischaemia and reperfusion (I/R) injury in animal models but has failed to translate in large clinical trials, possibly because experiments often use healthy animals lacking coronary atherosclerosis,or co-morbidities such as insulin resistance. We found RIC was ineffective in double-knockout SR-BI^ΔCTΔCT^;*Ldlr* KO mice that develop coronary atherosclerosis, or in insulin-resistant mice. However, UCL-TRO-1938, a phosphatidylinositol 3-kinase alpha activator that bypasses cell-surface receptors, was cardioprotective in these mice. The double-mutant SR-BI^ΔCTΔCT^;*Ldlr* KO strain may be a more clinically translatable mouse model of I/R. Furthermore, UCL-TRO-1938 is a promising cardioprotective drug as it remains effective in a mouse model with coronary artery disease.

## Supplementary Material

cvaf111_Supplementary_Data

## Data Availability

The data underlying this article will be shared on reasonable request to the corresponding author.
