# Supplementary figures and images for: An activator of phosphatidylinositol 3-kinase alpha restores cardioprotection from ischaemia/reperfusion injury in mice with coronary atherosclerosis or insulin resistance
Source: Cardiovasc Res. 2025 Jun 13;121(11):1722–33. doi: 10.1093/cvr/cvaf111 (PMC12477676; doi:10.1093/cvr/cvaf111)

**Supplementary figures**
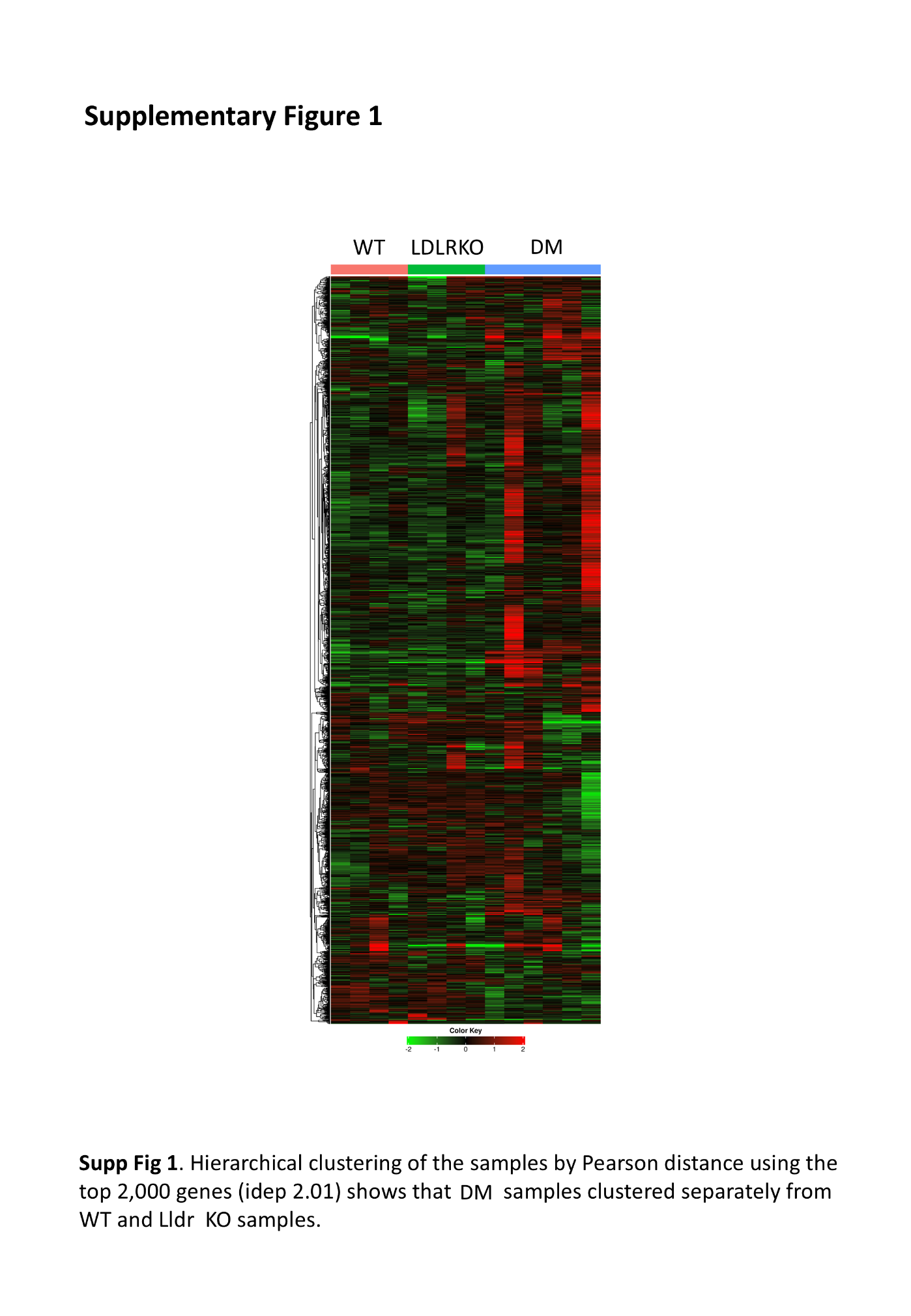

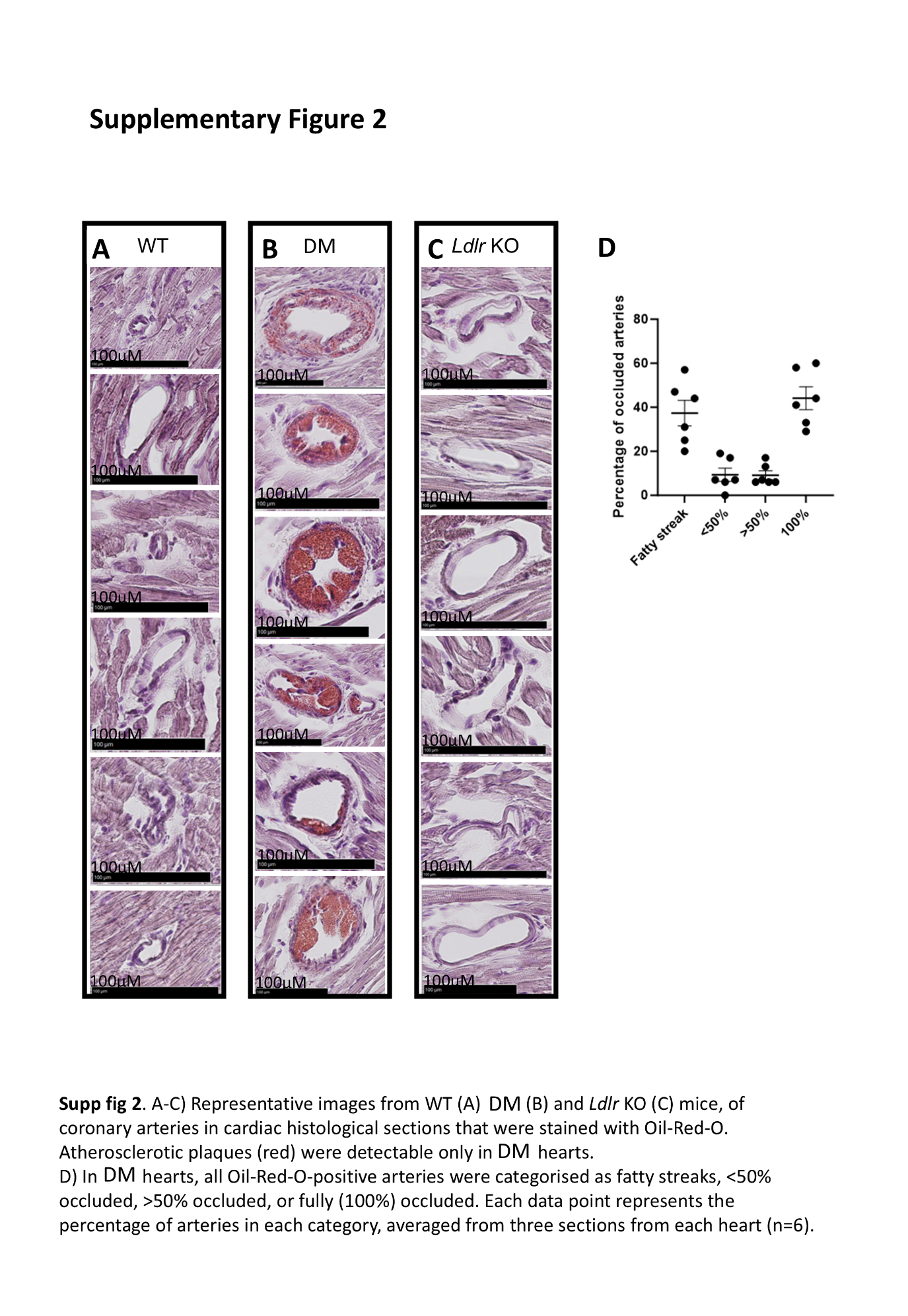
**
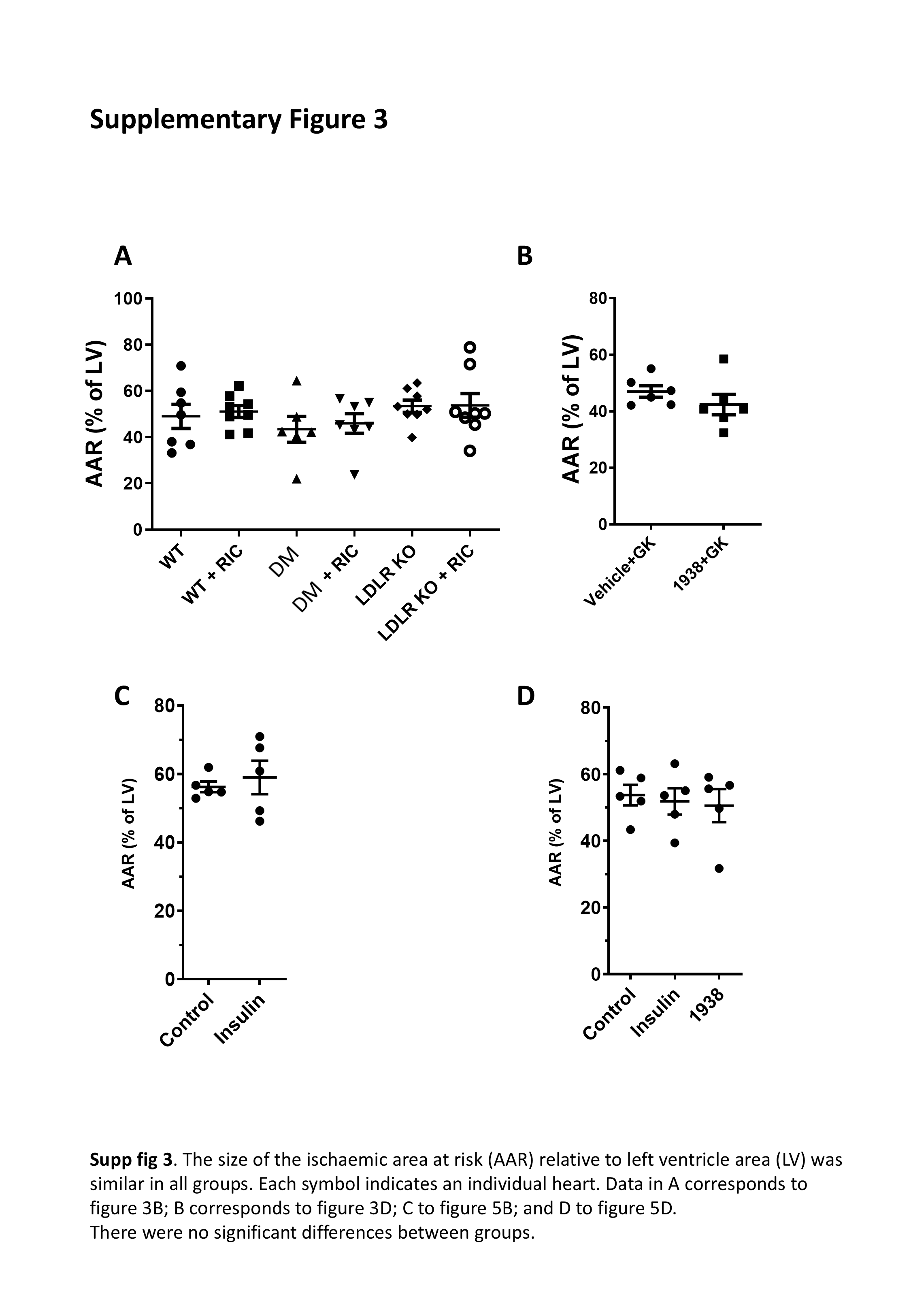

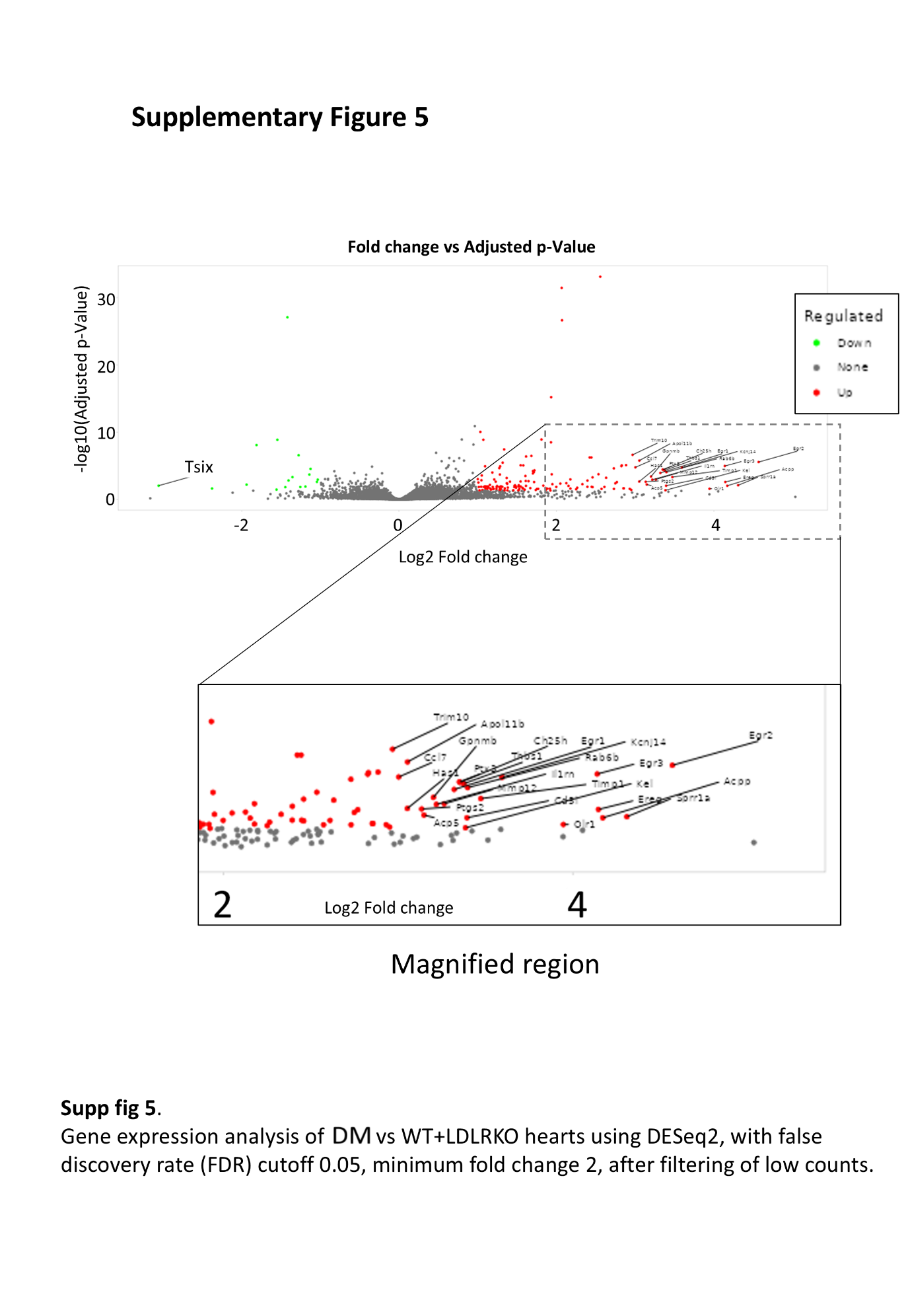

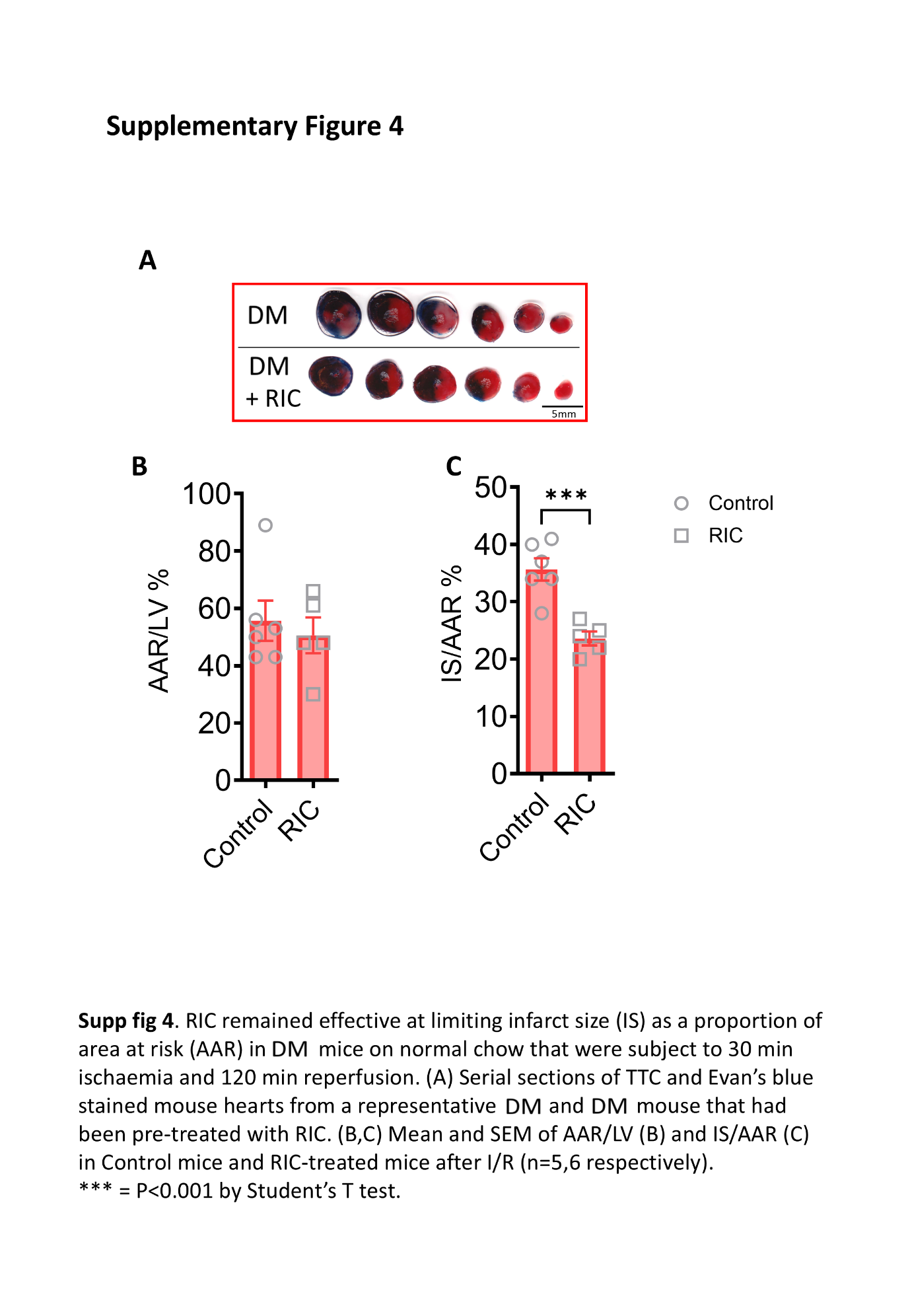
**

Supplement: cvaf111_Supplementary_Data [file cvaf111_supplementary_data.zip › SupplementaryfiguresRev2.docx]
